# Odor sampling strategies in mice with genetically altered olfactory responses

**DOI:** 10.1371/journal.pone.0249798

**Published:** 2021-05-03

**Authors:** Johannes Reisert, Glen J. Golden, Michele Dibattista, Alan Gelperin

**Affiliations:** 1 Monell Chemical Senses Center, Philadelphia, PA, United States of America; 2 Department of Basic Medical Sciences, Neuroscience and Sensory Organs, University of Bari “A. Moro”, Bari, Italy; 3 Princeton Neuroscience Program, Princeton University, Princeton, NJ, United States of America; Francis Crick Institute, UNITED KINGDOM

## Abstract

Peripheral sensory cells and the central neuronal circuits that monitor environmental changes to drive behaviors should be adapted to match the behaviorally relevant kinetics of incoming stimuli, be it the detection of sound frequencies, the speed of moving objects or local temperature changes. Detection of odorants begins with the activation of olfactory receptor neurons in the nasal cavity following inhalation of air and airborne odorants carried therein. Thus, olfactory receptor neurons are stimulated in a rhythmic and repeated fashion that is determined by the breathing or sniffing frequency that can be controlled and altered by the animal. This raises the question of how the response kinetics of olfactory receptor neurons are matched to the imposed stimulation frequency and if, vice versa, the kinetics of olfactory receptor neuron responses determine the sniffing frequency. We addressed this question by using a mouse model that lacks the K^+^-dependent Na^+^/Ca^2+^ exchanger 4 (NCKX4), which results in markedly slowed response termination of olfactory receptor neuron responses and hence changes the temporal response kinetics of these neurons. We monitored sniffing behaviors of freely moving wildtype and NCKX4 knockout mice while they performed olfactory Go/NoGo discrimination tasks. Knockout mice performed with similar or, surprisingly, better accuracy compared to wildtype mice, but chose, depending on the task, different odorant sampling durations depending on the behavioral demands of the odorant identification task. Similarly, depending on the demands of the behavioral task, knockout mice displayed a lower basal breathing frequency prior to odorant sampling, a possible mechanism to increase the dynamic range for changes in sniffing frequency during odorant sampling. Overall, changes in sniffing behavior between wildtype and NCKX4 knockout mice were subtle, suggesting that, at least for the particular odorant-driven task we used, slowed response termination of the odorant-induced receptor neuron response either has a limited detrimental effect on odorant-driven behavior or mice are able to compensate via an as yet unknown mechanism.

## Introduction

Breathing patterns of mice are complex and mice can dynamically and quickly change their breathing rates. Besides changing the respiratory rate, this also changes the frequency and duration with which olfactory receptor neurons (ORNs) are exposed to odorants carried by the inhaled air. Rhythmogenesis can be controlled voluntarily by afferent CNS input [1] and is also influenced by both odorant concentration and the behavioral context in which the odorant is presented [see e.g. 2–8]. Active olfactory exploration is often accompanied by an increase in breathing frequency (“sniffing”), the latter having been implicated in such diverse functions as directing odorant flow to different parts of the olfactory epithelium, increasing odorant flux to the olfactory epithelium, promoting discrimination ability, discovery of new odorants, adaptive filtering of olfactory information, encoding of olfactory information in the olfactory bulb and coordination of the olfactory system with other brain areas [2–4,9–15]. The change in breathing frequency from typically 2–5 Hz at rest to 5–10 Hz during a sniff bout in rats and mice can be triggered by the presentation of a novel odorant or in anticipation of an olfactory task with the increase in breathing frequency preceding the odorant presentation by approximately one sniff cycle [2,4,16,17]. An increase in breathing frequency is mainly achieved by shortening the exhalation phase of the sniff cycle, with the inhalation duration staying relatively constant [10]. 1–2 sniffs, or around 250 ms of odorant exposure, are sufficient to reliably distinguish odorants as shown by behavioral experiments in mice and rats [2,18–20].

Particularly in the behavioral regime where a single sniff or a few sniffs can provide sufficient information to allow odor identification or discrimination, it is important to understand the responses of ORNs within the first few 100 s of milliseconds of odor onset [21,22]. ORNs respond reliably with the firing of action potentials when first exposed to odorants on the first inhalation, and also do so to repeated stimulation as long as the stimulation frequency remains low. But even at repeated stimulations at around 5 Hz mouse ORNs begin to respond unreliably or do not generate action potentials at all [21], essentially applying a temporal low-pass filter to the stimulus pattern. The broad outlines of the odorant transduction mechanisms that underlie this behavior in ORNs are understood [22–26] including several of the essential proteins [27–30], metabolic [31] and second messenger pathways involved. Key regulators of gene expression required for development of ORNs [32,33] have also been identified. Binding of an odorant molecule to an odorant receptor in the ciliary membrane of an ORN leads to activation of a G protein, which then activates adenylyl cyclase 3, resulting in an increase in cAMP. Increased levels of cAMP open the olfactory cyclic nucleotide-gated (CNG) channel, resulting in an influx of Ca^2+^. The latter then activates an excitatory Ca^2+^-activated Cl^-^ channel (Anoctamin 2, Ano2) that carries the majority of the odorant-evoked current.

Understanding the links between these discrete transduction steps in ORNs and potential behavioral deficits produced by deletions of individual transduction steps can be complex. Deletion of certain transduction components, like the G protein components G_αolf_ or γ13, the CNGA2 channel subunit or adenylyl cyclase renders ORNs nonfunctional and mice behaviorally anosmic [34–37], while the deletion of other transduction components leads to more complex and more subtle behavioral alterations [29,38–47]. These more subtle alterations offer the possibility to discover how changes in olfactory transduction might alter olfactory behavior in general and sniff sampling in particular.

We focused on the role of K^+^-dependent Na^+^/Ca^2+^ exchanger 4 (NCKX4) in determining behavioral odorant detection, discrimination acuity and sniffing in an olfactory detection and discrimination task. NCKX4’s role is to remove Ca^2+^ that entered ORNs during the odorant response. This removal of Ca^2+^ is required to lower internal Ca^2+^ to its pre-stimulus levels, which allows the Ca^2+^-activated Cl^-^ channel to close and terminates the odorant-induced response. When NCKX4 is blocked or genetically ablated, the odorant-induced response is greatly prolonged due to the continued presence of a Cl^-^ current well beyond the end of odorant stimulation [44,48]. This prolonged current keeps the ORN depolarized to such an extent that voltage-gated Na^+^ channels become inactivated [49] and no new action potentials are generated when the ORN is stimulated again. Hence ORNs need a longer recovery period before the next stimulation is able to again generate action potentials and to signal odorant stimulation to the brain. As NCKX4 is only important in terminating the response, the early phase of the odorant response is very little affected [44]. The olfactory ability of NCKX4 knockout mice is impaired as they require a longer time to find a hidden piece of food [44]. Overall, this makes the NCKX4 knockout mouse an interesting model to study the effects of changes in ORN kinetics on behavior as well as potential compensatory mechanisms used in odor sampling strategies to compensate for the altered kinetics in the peripheral ORN response. We addressed this question by performing experiments to record the sniffing patterns of freely-moving control and NCKX4 knockout mice while they performed odorant-driven behavioral tasks [50,51].

## Materials and methods

All surgical procedures and handling of mice were approved by the Institutional Animal Care and Use Committee of the Monell Chemical Senses Center. Only male mice were used in this study. WT and NCKX4 knockout mice were derived from heterozygous breeders and genotyped following standard protocols [44]. Mice were of a mixed 129s6SvEV–C57BL/6 background. Mice were on a water-deprivation schedule before the experiments and were rewarded with small aliquots of water during the behavioral experiments.

### Acquiring breathing signals

We implanted mice with telemetric thoracic breathing sensors [PhysioTel TA11PA-C10, Data Sciences International (DSI) (St. Paul, USA)] to record their thoracic pressure and therefore their breathing frequency as previously described [17,50,52,53]. Mice were anesthetized with isoflurane and depth of anesthesia was tested with a toe pinch. To gain sensor access to the thoracic cavity, the sensor catheter was inserted along the serosal layer of the esophagus through the diaphragm. The main body of the transmitter was sutured to the abdominal wall. Potential postoperative pain was alleviated with subcutaneous injection of Buprenorphine (0.5–2.0 mg/kg). Following the conclusion of the study, mice were euthanized with CO_2_ followed by cervical dislocation.

A receiver platform (RPC-1) for wireless sensor signals was located below the experimental cage. It continuously recorded the pressure signal during the experiment and digitized the sensor signal with a commercial telemetry system (Matrix 3643, DSI). Data were sampled at 500 Hz and filtered at DC– 100 Hz. Custom-written MATLAB software was used to analyze the breathing signals. Band-pass filtering from 2–15 Hz removed baseline drifts and high frequency noise. Inhalation and exhalation peaks were detected using the Matlab function “findpeaks”. In two further steps, peaks and troughs that did not exceed a certain threshold (typically 3mmHg) were eliminated and when the frequency between two peaks exceeded 15 Hz only the larger peak (and larger trough) were retained. Finally, in a visual inspection cycle, spurious peaks and troughs were manually eliminated and missed peaks and troughs were manually added. In a final step, the peak and trough times were matched to the nearest peaks and troughs in the unfiltered data to determine the precise timings and thus the breathing frequencies. This approach yielded similar results when compared to simultaneously recorded nasal pressure signals [50]. S1 Fig shows the same sniff traces as in Fig 3 with peaks and troughs marked as detected by our algorithm.

### Odorant-driven behavioral experiments

Methods for training the mice on the odorant detection and discrimination task are described in detail in Reisert et al. [17] and are based on Slotnick & Restrepo [54]. A modified olfactometer [based on 55] with two ports was used, one odor delivery port and one to deliver water rewards [50]. IR beams crossing the entrance to the odor and water ports respectively monitored the animal’s entry and exit time for each port. Upon entry into the odor port a new trial began, and after a 0.5 s delay an odorant selected from one of eight odorant vials was delivered to the odor port for the duration of the mouse nose poke and terminated when the mouse withdrew from the odor port. The choice of the delivered odor and its behavioral relevance (Go, NoGo), and acquisition of time stamps (odor port in/out, water port in/out) were recorded and controlled using ABET software (Lafayette Instruments, Lafayette, USA). The odorants used were 1-propanol and eugenol diluted in filtered mineral oil at the vol/vol concentrations given below for each experiment.

Mice performed Go/NoGo tasks. Exposure to an odorant was rewarded with water (“go”, or S+) in the water port, while no odor (mineral oil control) was not (“NoGo” or S-). First, mice were trained to remain in the odor port for longer durations as longer sampling times improve their accuracy [18–20] for up to a total of 1.5 s. Once the task was learned, mice proceeded to the Go/NoGo paradigm at a propanol concentration of 10^−4^ vol/vol dilution. At this point, mice were allowed to freely choose the duration of time they wanted to remain in the odor port to sample the odorant.

Two different experiments were performed.

### Dose response experiments

We first addressed how odorant concentration altered sniffing behavior, sampling duration and odorant identification accuracy. Mice performed blocks of 20 trials, 10 of which were S+ and 10 were S- trials, presented in a random order with the limitation that not more than 4 trials of the same kind occurred in a row. For S+ trials, only 7 out of 10 trials were actually rewarded with water to familiarize the mice with the contingency that, especially during difficult trials using low odorant concentrations, events that were perceived as S+, might not be rewarded. Also, with intermittent reinforcement the response becomes more resistant to extinction [56,57], an issue that, again, is relevant during harder discrimination tasks.

For each test propanol concentration, three blocks of test trials were recorded. Subsequently, data derived from typically three blocks were analyzed and averaged for each mouse.

### Adaptation experiments

A second experiment addressed the effects of increasing concentrations of background odor against which the mice had to distinguish a fixed propanol concentration at 10^−4^ dilution (S+) vs the control mineral oil (S-) delivered to the odor port. To adapt ORNs of the mice to the background odor, the normally odor-free air that flows into the behavioral chamber was passed through a reservoir that was filled with various propanol concentrations as indicated below, similar to Kelliher et al. [58]. The control background odor was mineral oil. For each background propanol concentration and mouse, typically 1–2 blocks of test trials at 10^−5^ to 10^−3^ dilutions and 5–6 blocks at 10^−2^ dilution up to neat odorant were recorded and analyzed. For mineral oil as the background odorant concentration, 5–10 blocks were recorded.

We adopted a stringent protocol for both types of experiments (described in detail in [17]) to ensure that mice indeed used the odor delivered to the odor port to make their behavioral decisions, instead of other stimuli, e.g. sound or vibrational signals, emanating from the olfactometer.

The four behavioral outcomes are Hits = mouse receives a water reward to the S^+^ odor, False Alarm (FA), the mouse seeks a water reward to the S^-^ odor, Correct Rejection (CR) the mouse does not seek water to the S^-^ odor, Miss = failure to seek water reward to S^+^ odor. Misses were excluded from data analysis as only 0.75–3.5% of total trials were scored as Misses.

### Statistical analysis

In R environment (RStudio Team, 2015), dependent variables were investigated with linear mixed models (LMMs). LMMs were computed using the “lmer” function (lme4 package [59]). Predictors were different odor concentrations used (dose), genotype (WT vs KO) and behavioral outcome (Hits, CR, FA) and their interactions. Subject ID was introduced as a random effect.

To ensure that the random intercept and each predictor improved the model’s fit, the function “step” (lmerTest package, [60]) was used to perform automatic backward elimination. We started with a model that included all the predictors (both fixed and random) and then applied the “*step*” function that, by using the Akaike information criterion [61], first deletes the random part followed by backward elimination of the fixed part. In case the random intercept was removed, the “lm” function was used to fit linear models [59]. In summary, the “*step*” function removes those factors that did not significantly improve the model’s fit [60]. Finally, in order to get F statistics and p-values for the fixed effects of the models we ran ANOVAs using the lmerTest package [60].

Jamovi [The jamovi project (2019), https://www.jamovi.org] was used for repeated measure ANOVA. Post-hoc comparisons were performed as stated in the text or figure legends.

## Results

### Odor sampling and sniffing behavior when exposed to decreasing concentrations of odorant

**Fig 1:**
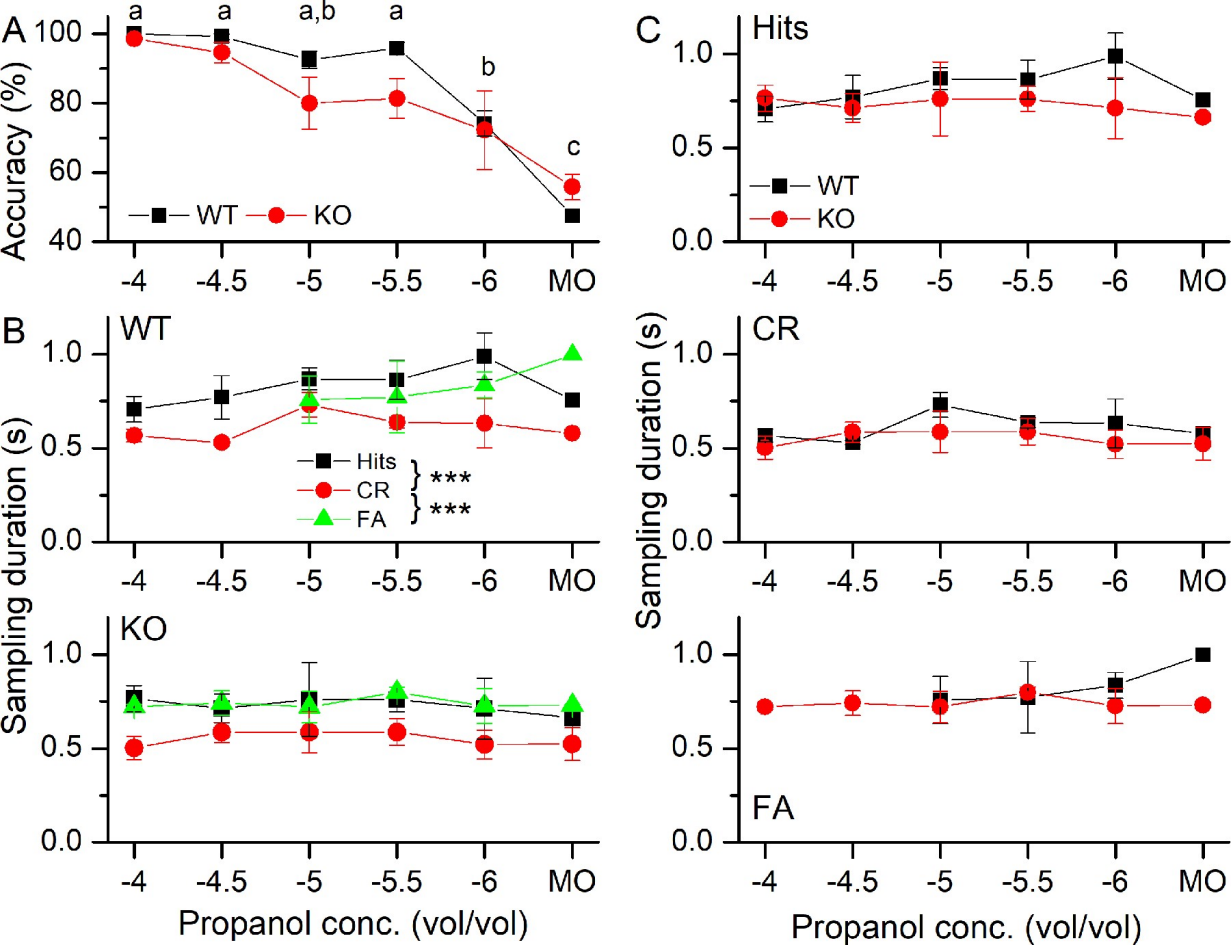
Accuracy and sampling behavior in a Go/NoGo odor-guided experiment in wildtype and NCKX4 knockout mice. **A** WT and NCKX4 KO mice were exposed to progressively lower propanol concentrations or the control odor mineral oil (MO) to establish their accuracy to distinguish between them. Accuracy has odor concentration as the main factor (F(9,10) = 8.63, p = 0.002), while no differences exist between genotypes. Same letters indicate the absence of significance between concentrations (post-hoc Tukey). **B** The odor sampling duration as a function of the odorant concentration and Hits, Correct Rejections (CRs) and, False Alarms (FAs) for the WT and the KO. The final model for sampling duration is a linear regression with mouse ID as a random effect and behavioral outcome as the main significant effect (F = 22.346, p = 1.711e^-8^). Post-hoc analysis revealed CRs being significantly less frequent than FAs (p < 0.0001) and Hits (p < 0.0001). **C** Comparison of the three behavioral outcomes between WT and KO. Data points are averages ± SEM of 3 WT and 3 KO mice. The x-axis displays the log of the odorant concentration.

**Fig 2:**
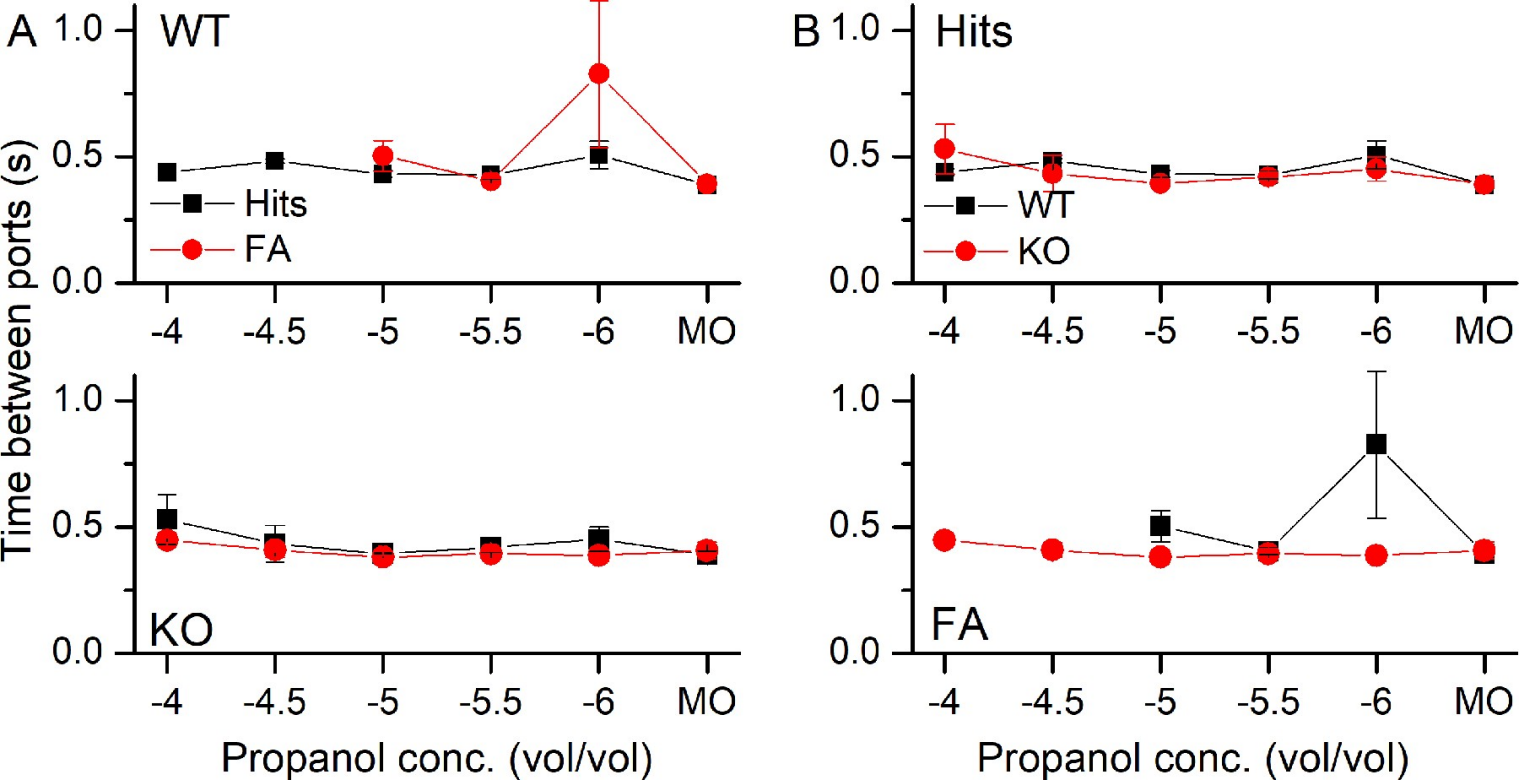
The odorant-dependent transition time between odor port and water port for WT and NCKX4 KO mice. **A** The time WT (top) and KO (bottom) mice spent between the odor and the water port following odorant sampling for Hits and FAs. **B** Comparison of Hits (top) and FAs (bottom) for WT and NCKX4 KO mice. The final model is a linear regression having behavioral outcome as the significant main factor (F = 5.056, p = 0.0308). Post-hoc analysis shows that time between ports is not different between Hits and FAs (p = 0.09, Tukey test). The model has a significant interaction between odorant concentration and genotype (F = 2.5318, p = 0.0461). A Tukey post-hoc test revealed that at a concentration of 10^−6^, the WT had a slower transition time than KO (p = 0.0083). Data points are averages ± SEM of 3 WT and 3 KO mice. The x-axis displays the log of the odorant concentration.

We first addressed how reliably mice could distinguish and detect decreasing odorant concentrations. Mice were trained to perform a Go/NoGo task to distinguish the water-rewarded S+ odorant propanol from the non-rewarded control solvent mineral oil (MO). Mice were also implanted with thoracic breathing sensors to simultaneously monitor their sniffing behavior. Mice were tested with decreasing concentrations to be discriminated from the control odor, MO (Fig 1A). At the high concentrations of 10^−4^ and 10^−4.5^ (vol/vol dilution of propanol), both wildtype (WT) and NCKX4 knockout (KO) mice, performed at or near perfect levels of accuracy with hardly any false alarms (FAs) or misses, meaning entering the water port when propanol was not supplied (FA) or not entering when odorant was presented (Miss). For both of these propanol concentrations, all three WT mice did not have any FAs (see “missing” FA data points in Fig 2), while one of the three KO mice did not have any FAs at these two concentrations. Further lowering the propanol concentration began to reduce the accuracy of determining the presence of the odor, but only at 10^−6^ and MO as the S+ stimulus did the accuracy drop significantly compared to the higher odorant concentrations. Repeated measure ANOVA showed odor concentration as the main factor with no difference between the genotypes. This suggests that altering the termination kinetics of the odorant response alone does not reduce the ability of mice to distinguish odorant concentrations during this particular odor-discrimination task.

Mice were exposed to the odorant for as long as they chose to remain with their noses in the odor port. As mice can improve their accuracy by sampling odorants for longer [20], we asked if WT or KO mice displayed different sampling durations associated with the three behavioral outcomes: Hits, correct rejections (CRs) and FAs. Misses were not included in the analysis, since overall Misses were sporadic and rare (see Methods and materials). In both the WT and the KO (Fig 1B), mice sampled for around 0.75 s for Hits across all odorant concentrations, but did so for a significantly shorter duration, for only around 0.5 s, for CRs. The sampling durations of FAs were more closely aligned with Hits and were significantly different from CRs. We also compared Hits, CRs and FAs between WT and KO directly (Fig 1C) and found them to be similar. Thus, mice did not seem to choose different sampling durations when NCKX4 was missing from their ORNs.

An additional aspect of the experimental setup is that mice have to physically transition from the odor port to the water port, a distance of 4 cm), to try to receive a water reward, which occurs for Hits and FAs. This transit time reflects the duration it takes a mouse to make a decision on the odor concentration and implement the motor response to move to the water port. Fig 2A compares Hits and FAs in WT and KO mice, while Fig 2B compares the WT and the KO for Hits and FAs. Note the aforementioned lack of FAs for 10^−4^ and 10^−4.5^ in the WT. Linear regression analysis revealed that Hits and FAs are a main factor and statistically different, although post-hoc analysis showed that the time between ports is not different between Hits and FAs (p = 0.09, Tukey test). Odor concentration and genotype showed a significant interaction and post-hoc analysis showed that at 10^−6^ the WT had a slower transition time compared to the KO (p = 0.0083, Tukey test).

**Fig 3:**
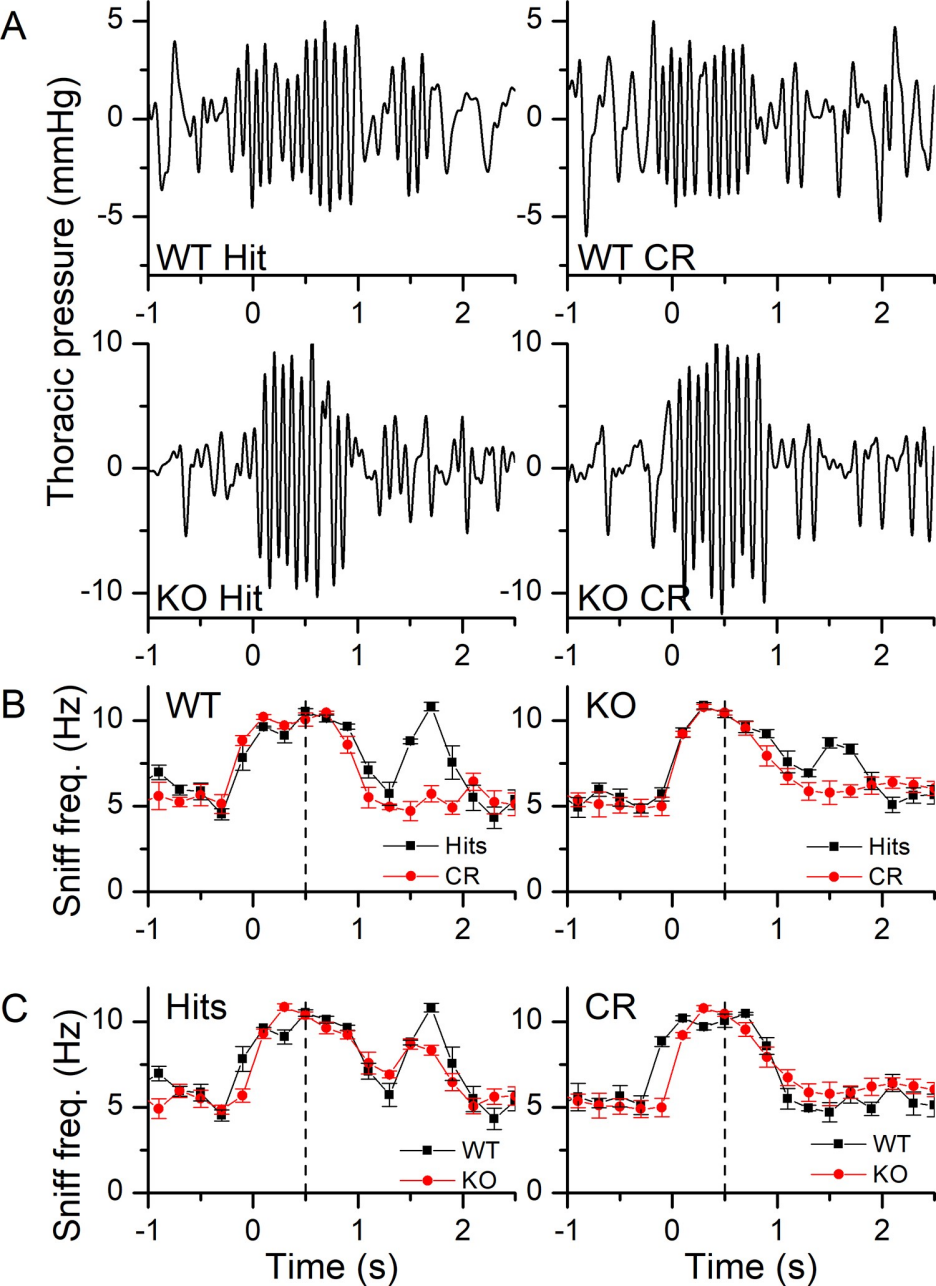
Recordings of thoracic pressure changes during odorant sampling of different odorant concentrations. **A** The thoracic pressure during a Hit (left) and a CR (right) trial in a WT and a KO mouse when exposed to 10^−4^ propanol. Time 0 marks the nose poke into the odor port and the start of the trial. Odorant delivery occurred at 0.5 s. **B** The sniff frequency of a WT (left) and a NCKX4 KO (right) mouse when tested with 10^−4^ propanol or mineral oil odor. **C** Comparison of Hits (left) and CRs (right) between WT and KO mice. Sniff frequencies were binned in 0.2 s bins according to their behavioral outcome and averaged (mean ± SEM) across trials of one block of 20 trials. Vertical dashed line indicates odorant onset.

To determine how mice sniff while odor sampling, we monitored thoracic pressure changes while mice performed the Go/NoGo task. Representative recordings of the thoracic pressure changes are shown in Fig 3A for a WT and a KO mouse, sniffing 10^−4^ propanol and correctly responding with a Hit (left) or being exposed to MO, the control odor, and responding correctly with a CR (right). Prior to entering the odor port mice sniffed at around 5 Hz, but they increased their sniffing to 10 Hz upon entering the odor port at t = 0 s, even before they were exposed to odorants at t = 0.5 s. Analysis of the time course of the change in sniff frequency shows that in the WT mouse the high sniff frequency was maintained for a longer duration during Hits compared to CRs (Fig 3B, left, and also Fig 5 for a more detailed analysis), with Hits also displaying a rebound to higher sniff frequencies when entering the water port. The KO (Fig 3B, right) had a similar sniff pattern compared to the WT with the aforementioned faster drop in sniffing frequency for CRs compared to Hits. Fig 3C compares Hits and CRs for WT (left) and KO (right). See also S1 Fig, which shows the same sniff traces as in Fig 3A with peaks and troughs marked.

**Fig 4:**
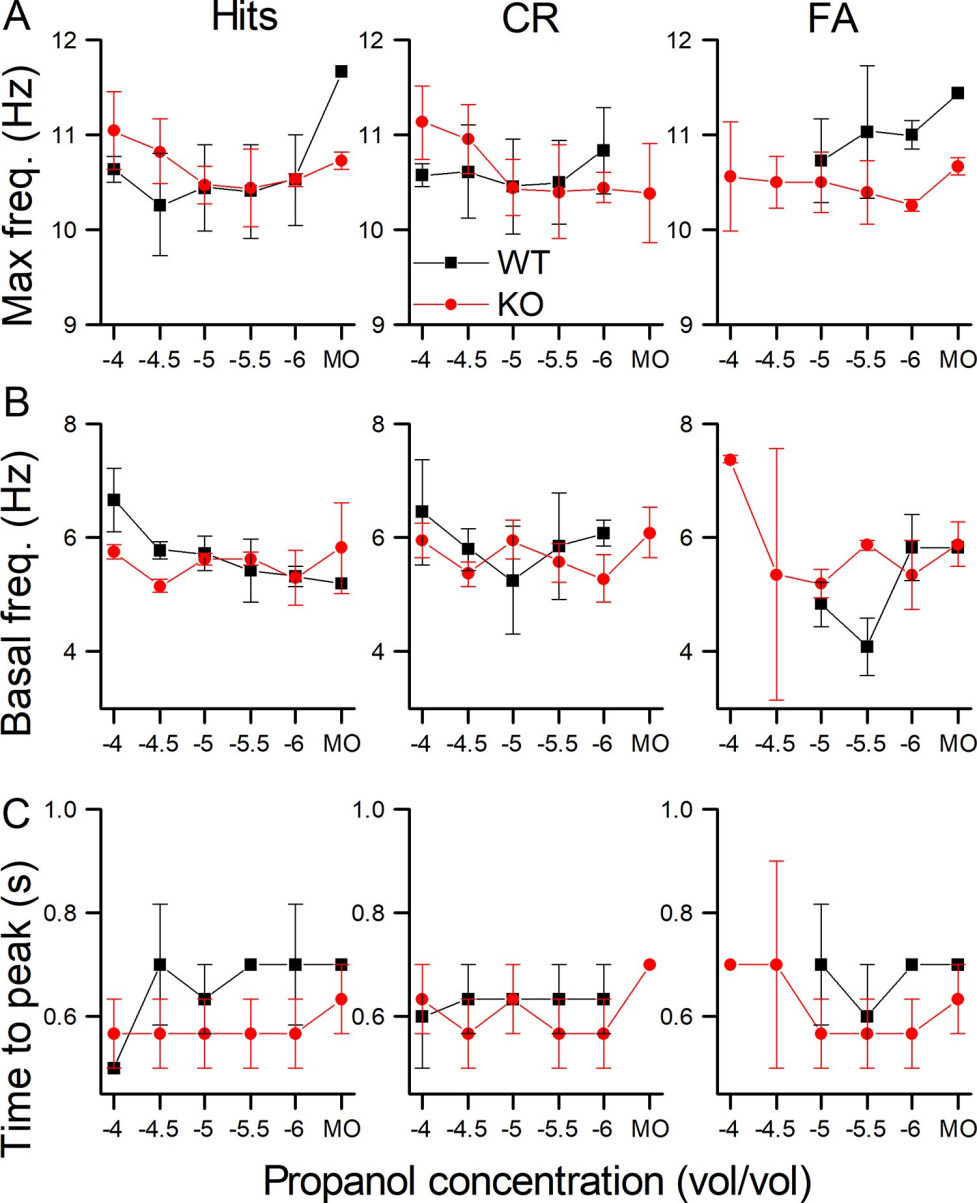
Parameters of sniff responses during odorant sampling. **A** Peak sniff frequency during odorant sampling for WT and NCKX4 KO mice for Hits, CRs and FAs. **B** Baseline sniff frequency averaged across all sniffs from -1 to 0 s. **C** Time to reach maximal sniff frequency calculated from the odorant onset at 0.5 s. Basal frequency and Max frequency had odorant concentration as the only term remaining in the model and it is significant as the main effect (F = 2.64, p = 0.03 and F = 2.8618, p = 0.03 respectively). Data points are averages ± SEM of 3 WT and 3 KO mice. The x-axis displays the log of the odorant concentration.

We quantified the sniff frequency response by determining the maximal frequency, the basal frequency (calculated as the average sniff frequency during the 1 s before entering the odor port) and the time to reach maximal sniff frequency (see Fig 4A to 4C respectively) for Hits, CRs and FAs. For these three parameters, their dependence on genotype was relatively small. The odorant concentration, the basal frequency and the maximal sniff frequency odorant concentration remained as the only significant main effects. The time to peak proved not to be amenable to analysis with either a linear mixed regression or a linear regression.

**Fig 5:**
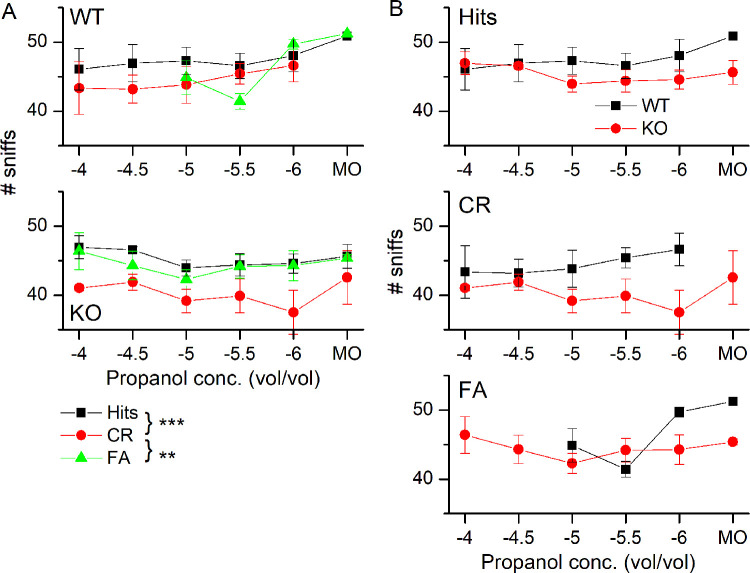
Number of sniffs taken during odorant sampling. **A** Comparison of the total number of sniffs taken during the five 200 ms bins centered on 0.5 to 1.3 s in WT (top) and NCKX4 KO (bottom) mice for Hits, CRs and FAs. **B** Comparison of Hits, CRs and FAs between the WT and the KO. The number of sniffs has behavioral outcome as the only predictor in the final model with it being the main significant effect (F = 13.97, p = 6.127e^-06^), and post-hoc comparisons (Tukey) show that the number of sniffs for CRs is significantly less than that of FA (p = 0.0099) and Hits (p < 0.0001). Data points are averages ± SEM of 3 WT and 3 KO mice. The x-axis displays the log of the odorant concentration.

We analyzed the sniffing behavior in more detail to quantify the observed differences in high frequency sniff durations. To do so, we summed all sniffs taken in the five time bins (duration of time bin = 0.2 s) centered on 0.5 s to 1.3 s and compared them for WT and KO mice (Fig 5A). Comparing the number of sniffs between Hits and CRs showed that in the presence of odorant exposure, mice took fewer sniffs in the 1 s after odorant exposure began, mirroring their shorter sampling duration during CR trials (see Fig 1). During FAs, the number of sniffs taken resembled those taken during Hits and were significantly different from CRs. In Fig 5B, the same data were replotted to compare Hits, CRs and FAs between WT and KO animals. While a trend might be apparent that the numbers of sniffs taken in the WT is higher than in the KO, this trend was not significantly different.

### Odor sampling and sniffing behavior in the presence of background odorants

**Fig 6:**
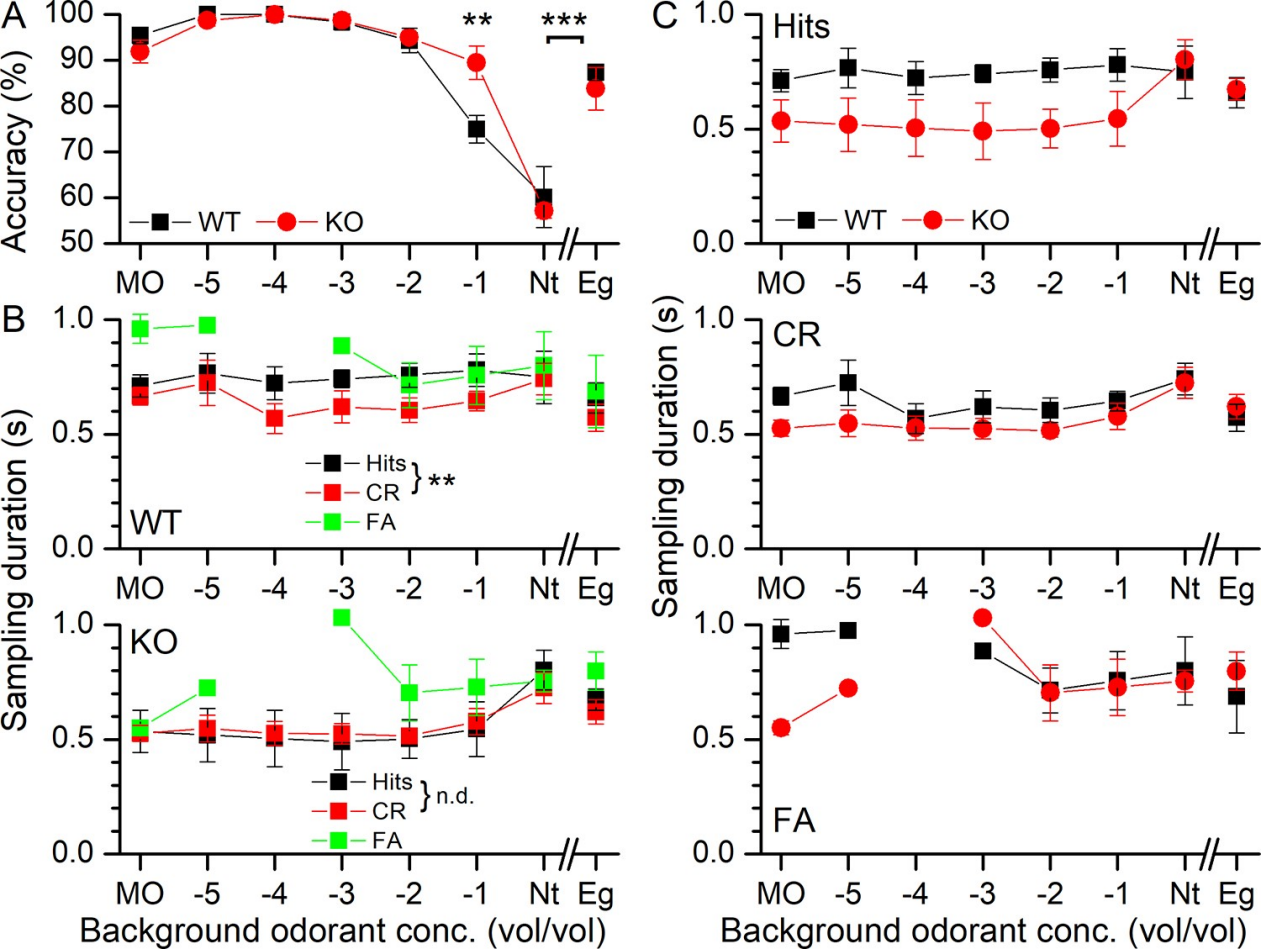
Accuracy and odor sampling duration in the presence of background odorants. **A** WT and NCKX4 mice performed a Go/NoGo task to distinguish 10^−4^ propanol from the control odor of mineral oil (MO) in the presence of increasing background concentrations of propanol up to the highest concentration of undiluted propanol (neat, Nt) and also undiluted eugenol (Eg). Accuracy with background odor present shows a significant interaction between background odor concentration and genotype. Post-hoc analysis revealed that at a concentration of 10^−1^ the odor identification accuracy is different between WT and KO with the KO having higher accuracy (p = 0.01). **B** The odor sampling duration as a function of the background odorant concentration for Hits, CRs and FAs for WT and KO. **C** Comparison of Hits, CRs and FAs between WT and KO. For sampling duration the final model has a significant 3-way interaction between behavioral outcome, genotype and background odorant concentration (F = 1.98, P = 0.03). Due to missing data points for FAs, FAs could not be estimated in post-hoc (Tukey) comparisons while for CR and Hits sampling duration in CR is shorter than for Hits in the WT (p = 0.0093) while no differences were seen between CR and Hits in the KO (p = 0.8393). Sampling durations in the CRs are very similar for WT and KO while Hits tend to be longer in WT (p = 0.0582). Data points are averages ± SEM of 3 WT and 4 KO mice. The x-axis displays the log of the odorant concentration. Round brackets indicate differences across behavioral outcome, while square brackets denote evaluation across concentration (or odor).

Ca^2+^ clearance by NCKX4 knockout mice from their olfactory cilia is required for ORNs to recover from odorant adaptation and to be able to fire action potentials again [21,44,48]. We thus addressed how mice would sample odorants in the presence of increasing concentrations of a background odorant. For this experiment, the odorant concentration to which mice were exposed in the odor port was kept at 10^−4^ propanol for all experiments, while the odorants blown into the behavioral chamber were increasing concentrations of propanol and, as a control, also the odorant eugenol. At the lower background concentrations from the control mineral oil (MO) up to 10^−2^ both the WT and the KO performed at near perfect accuracy (Fig 6A). At 10^−1^ background concentration, the accuracy of the WT mice dropped to about 70% and to near chance at neat propanol (Nt). Surprisingly, the KO mice continued to perform at higher accuracy at the background concentration of 10^−1^, but also dropped to near chance at Nt. When neat eugenol (Eg) was used as the background, both WT and KO again performed at a high accuracy, suggesting that it is not the high background odorant concentration per se that leads to the near chance performance at Nt, but instead the specific adaptation to the test odorant.

Next, we evaluated the time mice chose to sample the odorant in the odor port (Fig 6B) in order to make their odor identification decision. WT mice took odor samples for a relatively constant duration across the range of applied odor backgrounds, with Hits associated with significantly longer sampling times compared to CRs. FAs varied more across the odor backgrounds and probably due to missing data points (when mice did not have FAs when performing the test) statistical significance could not be estimated in the post-hoc tests. In contrast, the KO sampling durations for both Hits and CR were similar and constant across the lower background concentrations but increased at the two neat background concentrations (Nt, Eg). FAs again showed a more variable sampling duration, particularly at the lower background concentrations but with a consistent sampling duration at the higher background concentrations. In contrast to the WT, KO sampling durations during Hits and CRs were not different from each other. We also directly compared the sampling durations for Hits, CRs and FAs between the WT and the KO (Fig 6C). For Hits, sampling durations for the WT were longer compared to the KO, but this did not reach statistical significance (p = 0.058), while sampling durations during CRs were more similar as were FAs at the higher backgrounds.

**Fig 7:**
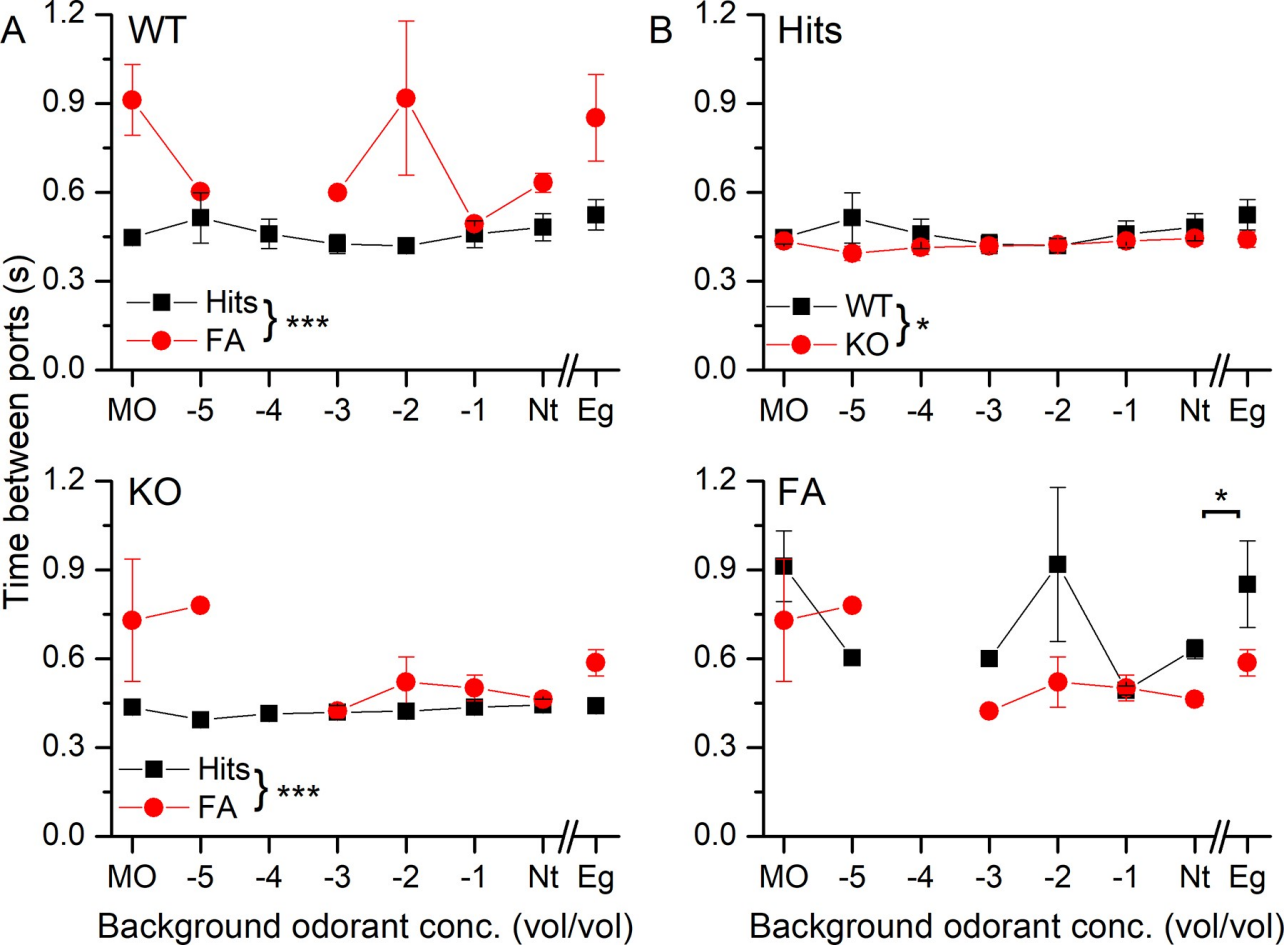
The odorant-dependent transition time between odor port and water port for WT and NCKX4 KO mice in the presence of background odorants. **A** The transition times for WT (top) and KO (bottom) mice between the odor and the water port following odorant sampling for Hits and FAs. **B** Comparison of Hits (top) and FAs (bottom) for WT and KO mice. The time between ports has as a final model a linear regression that has genotype (F = 5.7642, p = 0.02) and type (F = 24.0075, p = 5.843e^-06^) as significant effects. Tukey post-hoc analysis revealed that WT has a slower transition between the ports (p = 0.02) than KO. Also, overall, the time between ports is higher in the FA that in Hits (p = 1e^-06^). Repeated measures ANOVA showed that when considering transition times between ports in Nt vs Eugenol background odor trials there is a significant interaction between behavioral outcome and genotype (F = 7.13, p = 0.044) and behavioral outcome and background odor (F = 10.14, p = 0.024). Time between ports is different between Nt and Eg only for FAs (p = 0.046). Also, Eg FA is different from Nt (p = 0.006) and Eg for Hits (p = 0.001). Data points are averages ± SEM of 3 WT and 4 KO mice. The x-axis displays the log of the odorant concentration. Round brackets indicate differences across behavioral outcome or genotype, while scare brackets denote evaluation across concentration (or odor). Nt is neat propanol and Eg is neat eugenol.

Fig 7A compares the time between odor and water ports in the WT (top) and the KO (bottom). In both cases during Hit trials, the transition time stayed relatively constant at around 450 ms. WT and KO mice took longer times during FAs to move to the water port. Comparison of Hits and FAs between WT and KO (Fig 7B) revealed that the WT took a longer time between the ports for both Hits and FAs. Comparison of Nt vs Eg backgrounds showed that these transition times were significantly longer during FAs in the WT compared to the KO.

**Fig 8:**
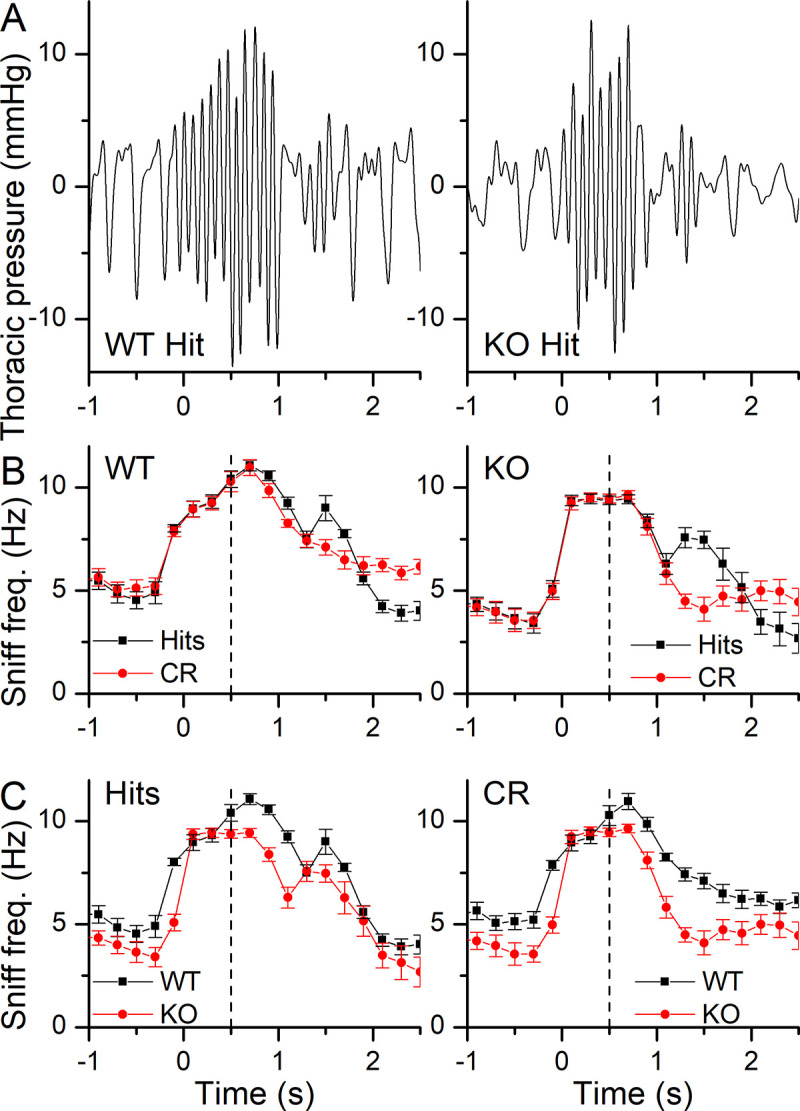
Recordings of thoracic pressure changes during odorant sampling at different background odorant concentrations. **A** Thoracic pressure changes during a Hit trial of a WT (left) or a NCKX4 KO (right) mouse when tested with 10^−4^ propanol delivered in the odor port and with the background odor of the control mineral oil. Time 0 marks the nose poke into the odor port and the start of the trial. Odorant delivery began at 0.5 s. **B** The sniff frequency of Hits and CRs of a WT (left) and a KO (right) mouse when tested with 10^−4^ propanol vs mineral oil delivered in the odor port in the presence of mineral oil as the control background odorant. **C** Comparison of Hits (left) and CRs (right) between WT and KO mice. Sniff frequencies were binned in 0.2 s bins according to their behavioral outcome and averaged (mean ± SEM) across trials of one block of 20 trials. Vertical dashed line indicates odorant onset.

**Fig 9:**
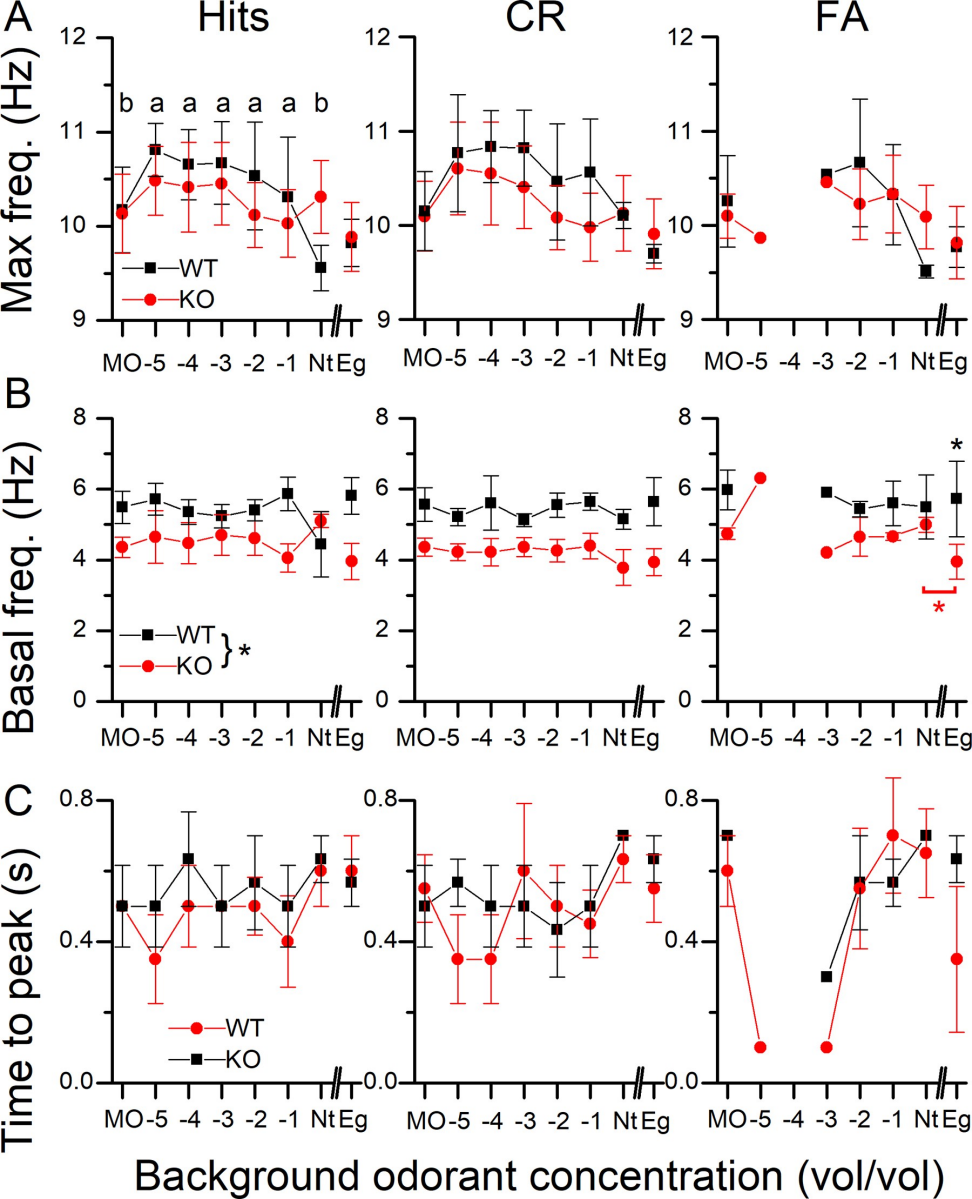
Parameters of sniff responses in the presence of background odorants. **A** Peak sniff frequency during odorant sampling for WT and NCKX4 KO mice for Hits, CRs and FAs. **B** Baseline sniff frequency averaged across all sniffs from -1 to 0 s. **C** Time to reach maximal sniff frequency calculated from the odorant onset at 0.5 s. Max frequency has a significant interaction between genotype and background odorant (F = 3.2, p = 0.006). Also, only in the WT the Max frequency in Nt is significantly lower that of the other concentrations except MO (same letters indicate lack of significance). Max Frequency in MO was different from -5, -4 and -3 in the WT, no differences were seen for the KO. Basal frequency has genotype and behavioral outcome as main effects (F = 9.07, p = 0.02 and F = 3.7, p = 0.027 respectively) with WT different from KO (p = 0.03) and CRs being different from FA (p = 0.028). Time to peak has behavioral outcome and background odor concentration as significant interaction (F = 2.1867, p = 0.021). Data points are averages ± SEM of 3 WT and 4 KO mice. The x-axis displays the log of the odorant concentration.

**Fig 10:**
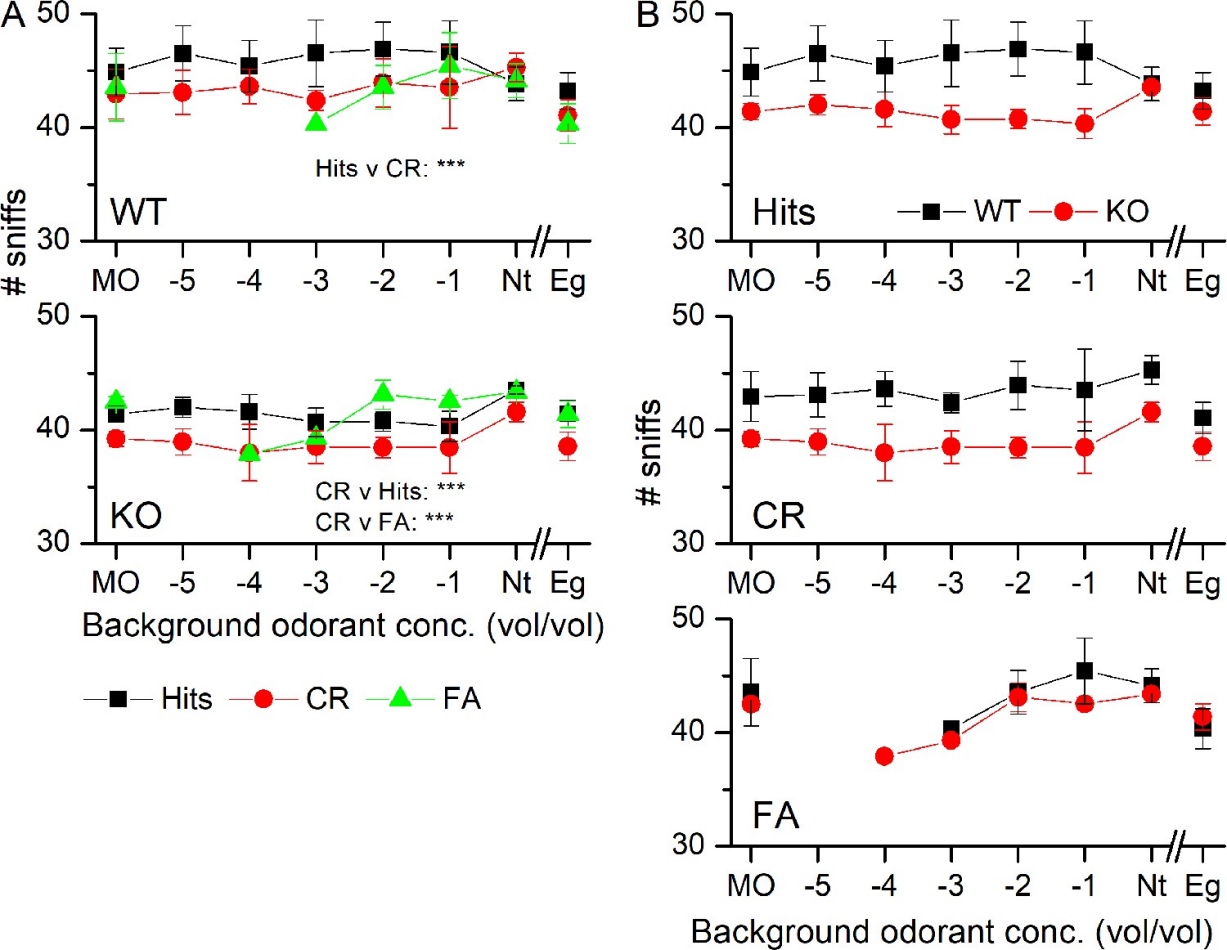
Number of sniffs taken in the presence of background odorants. **A** Comparison of the total number of sniffs taken during the five 200 ms bins centered on 0.5 to 1.3 s in WT (top) and NCKX4 KO (bottom) mice for Hits, CRs and FAs in dependence of the background odorant. **B** Comparison of Hits, CRs and FAs between the WT and the KO. The number of taken sniffs has a significant interaction between behavioral outcome and genotype (F = 4.4262, p = 0.014). Post-hoc comparisons (Tukey) show that while in the WT CR are different only from Hits (p = 0.0084), in the KO CR are different from FA (p = 0.0001) and Hits (p = 0.0002). No significant differences are detected between WT and KO. Repeated measures ANOVA. Nt versus Eg show are overall different (F = 9.727, p = 0.036). Data points are average ± SEM of 3 WT and 4 KO mice. The x-axis displays the log of the odorant concentration.

We also recorded the sniffing behavior in the behavioral odor-adaptation paradigm. Thoracic pressure changes are shown in Fig 8A for WT and KO Hits (right and left panel respectively) with MO being the background control odorant. Mice were breathing at their basal rate of around 5 Hz and upon entering the odor port quickly increased their sniffing frequency to 10 Hz, with the odorant being delivered at 0.5 s after nose poke onset. During Hits, mice maintained their elevated sniffing rate for longer at a high frequency (see Fig 8B and also Fig 10) compared to CRs and also showed a secondary rebound to a higher sniff frequency when entering the water port, which was mostly absent in CRs. Fig 8C compares Hits and CRs between the WT and the KO. A more quantitative analysis of the sniffing frequency responses is provided in Fig 9 for the maximal sniff frequency, the time to reach maximum sniff frequency and the basal sniff frequency prior to entering the odor port. For the maximal frequency (Fig 9A), the WT appears to reach overall higher peak sniff frequencies compared to the KO and has a significant interaction between genotype and background odorant concentrations. In the WT, the peak sniff rate at Nt background concentration is significantly lower than all other backgrounds except MO. Comparison of the basal frequency (Fig 9B) between WT and KO showed that KO mice had a significantly lower basal sniff frequency, reduced by around 1 Hz, compared to WT mice across all behavioral outcomes. Only when comparing Nt and Eg, for the KO, were basal sniff rates significantly higher for Nt compared to Eg. Time to peak has behavioral outcome and background odorant concentration as a significant interaction. Post-hoc tests did not reveal further differences.

Lastly, we investigated the numbers of sniffs mice took in this behavioral paradigm (Fig 10). For both the WT and the KO, mice sampled with more sniffs during Hits compared to CRs. In the KO, sniffs during FAs were also significantly higher compared to CRs (Fig 10A). In a comparison of the three behavioral outcomes between WT and KO (Fig 10B), the KO appeared to take fewer sniffs, but the difference was not significant. Comparing number of sniffs between Nt versus Eg, showed that CRs are different from Hits and FAs.

## Discussion

Mice can dynamically and voluntarily alter the frequency with which they breathe and therefore stimulate their ORNs in the nasal cavity [62]. Under normal conditions mice are obligate nose breathers [63] so nasally-inspired air carries volatile odorants into the tortious nasal turbinates and over the nasal epithelium. We set out to address how the kinetic response properties of ORNs might contribute to selection of a sniffing strategy by monitoring the breathing/sniffing frequency of mice while performing odorant-driven behavioral tasks. We used a knockout of NCKX4 as ORNs that lack this transporter have greatly prolonged odorant responses, while maintaining the same activation kinetics [44]. When these mice are challenged to find a buried food pellet, the KO mice require substantially longer times to locate the food compared to WT mice, indicating that mice perform poorly when challenged with this odorant-driven task. As such we were surprised that, performing a Go/NoGo task, KO mice performed as well as (dose-response experiments) or even better than (background adaptation) the WT mice in selecting the correct odor-guided behavior (Figs 1A and 6A respectively). As mentioned above, action potential firing in ORNs, and hence the information sent to the olfactory bulb, is determined by the onset of the odorant-induced response and is unchanged in the NCKX4 KO mice for a single odorant exposure, but not when ORNs are stimulated repeatedly, in which case, action potential generation is greatly suppressed during sniffs 2 –N. [44]. Given that mice might only need a single sniff, at least for easier discrimination tasks, to determine the identity of an odorant, one could suggest that, since the response to a single odorant exposure is the same in WT and NCKX4 KO mice, this may explain why the KO can maintain high accuracy during the dose response experiments. This argumentation is less likely to hold for the second set of experiments when mice were exposed to background odorants. In this case, ORNs that lack NCKX4 should be in a constant and prolonged state of adaptation [44,48] with a large reduction in signaling to the olfactory bulb. Yet, behaviorally, KO mice performed as well or even better compared to WT mice for reasons as yet unknown. As NCKX4 KO mice performed poorly in a food-finding task, one potential difference is that the food-finding task is an innate task, while in our experiments mice were trained extensively to perform the Go/NoGo task. The extensive training on the Go/NoGo task could allow the development of learned compensatory strategies over time that remain to be found (see also below).

Innate behaviors are encoded by hardwired neuronal circuitry that may be more sensitive to genetic changes e.g. gene deletion and their alteration in ORN physiology. A similar dichotomy has been observed in Ano2 KO mice where mice lacking the Ca^2+^-activated Cl^-^ channel were slower in performing a food-finding task while they performed as well as the WT in a Go/NoGo task [40,42,43]. This might be due to the fact that hardwired circuits are less plastic and may be more sensitive to perturbations in the organization of the olfactory system. On the other hand, Go/NoGo tasks involve higher degrees of neuronal plasticity that may compensate for the lack of transduction proteins. For example, corticofugal projections to the OB could modulate early olfactory signaling by inhibiting OB output neurons, thereby dynamically gating sensory throughput to the cortex. This could make the OB and higher olfactory centers the neural loci that govern these kind of tasks [64–66]. In particular, processing in olfactory areas downstream of the OB can occur on very fast timescales. We speculate that even with altered ORN response kinetics but still on a faster timescale compared to higher centers, peripheral responses may still have little influence on decision making and sniffing behavior. Also note a recent paper by Blount & Coppola [8] that suggests that supervised learning as in the Go/NoGo task employed here, can drive plasticity in higher centers to improve behavioral decisions and that behavioral parameters, e.g. odor sensitivity, are not simply a readout of peripheral ORN inputs.

As we reported previously [17], WT mice remained in the odor port and therefore sampled the odorant for longer times during Hit trials compared to CRs, which we again saw here for both the dose response and the background odorant adaptation experiments. This pattern was also seen for the KO in the dose response experiments, but not in the background odorant experiments where odorant sampling duration was different for Hits, but the same for CRs (Fig 6B). Thus, it appears that the presence of a background odorant drives both WT and NCKX4 knockout mice to use similar sampling strategies for CRs. Interestingly, this same pattern is not reflected in the numbers of sniffs taken, where in both the WT and the KO mice fewer sniffs are taken during CRs compared to Hits (Fig 10A). Intuitively, one might assume that the sampling duration and the number of sniffs co-vary. At least in NCKX4 KO mice in the presence of background odorants, it might not be the sampling duration per se that determines the number of sniffs taken. Alternatively, it might be that sniff patterns during odorant sampling are more “hardwired” independent of ORN response kinetics while sampling duration might be more malleable. In the background odorant experiments, but not in the dose response experiments, KO mice had a lower basal sniff rate prior to entering the odor port. This could increase the dynamic range with which the mice can alter their sniffing frequency following entry into the odor port and as such alter adaptive filtering of ORNs [4,21]. The reason for this change in basal sniff rate in the background odorant experiments is unclear. One possibility is that the mice are aware of which task they are about to perform and/or that even the mineral oil “background” odor concentration and even very low odor concentrations inform the mice that they will perform the background odorant task. And they, preemptively, alter their sniffing behavior accordingly.

Interestingly, mice that expressed the acetophenone M71 OR in 95% of all ORNs could perform a Go/NoGo task equally well compared to the control mice in a discrimination task with acetophenone as background odor [67]. In this situation acetophenone as the background odor would fully adapt the entire system but mice seemed to learn how to ignore this noisy background and complete the task with high accuracy. This might be a situation akin to the NCKX4 KO mice in the presence of a background odor.

Recent theoretical work has emphasized the potential importance of information conveyed in the first sniff and the elicited responses of mitral/tufted cells most sensitive to sensory cell input conveyed in the first sniff [68,69]. The experimental work supporting this “primacy code” relies on measurements in head-fixed mice which may or may not make perceptual decisions in a way similar to freely-behaving mice [70,71] as well as novel analytical methods applied to simultaneous multi-neuron recordings [72].

The behavioral test involving finding a buried food pellet revealed a deficit of NCKX4 mice in the times taken to discover the buried food pellet [44]. Interestingly this test involved a single 200 s trial per day, terminated when the mice discovered the food pellet. The NCKX4 knockout mice showed minor improvements over five days of testing and were consistently slower a localizing the buried food pellet than the control WT mice (cf Fig 6D in [44]). No explicit training was given prior to the test sessions. This is in contrast to the extensive training given to the mice in the two-port olfactometer used in the experiments reported here, thus providing ample time for adjustments in olfactory bulb circuitry as both WT and NCKX4 mice learned the odor identification and discrimination tasks in the olfactometer. There is a large literature documenting changes in M/T cell responses during odor learning, as recently summarized in [62]. As such, it would be interesting to address if and how bulbar circuitry might be altered in NCKX4 knockout mice as a potential compensatory measure against changes in peripheral ORN signaling.

## Supporting information

S1 Fig Recordings of thoracic pressure changes and detection of sniff peaks and troughs. The same sniff traces as presented in Fig 3A during a Hit (left) and a CR (right) trial in a WT and a KO mouse when exposed to 10^−4^ propanol. Time 0 marks the nose poke into the odor port and the start of the trial. Odorant delivery occurred at 0.5 s. Green and red circles mark peaks and troughs as detected with our custom-written software. Note that some circles are offset in relation to the apparent peaks here. Final time values were determined using the unfiltered data (as supposed to the filtered data shown here) to avoid any time shifts caused by the filtering of the data. For display purposes, the pressure values of the filtered data were used to tie the data points to the shown filtered traces.
(TIF)
